# Automotive System for Remote Surface Classification

**DOI:** 10.3390/s17040745

**Published:** 2017-04-01

**Authors:** Aleksandr Bystrov, Edward Hoare, Thuy-Yung Tran, Nigel Clarke, Marina Gashinova, Mikhail Cherniakov

**Affiliations:** 1 School of Engineering, University of Birmingham, Birmingham B15 2TT, UK; e.g.hoare@bham.ac.uk (E.H.); m.s.gashinova@bham.ac.uk (M.G.); m.cherniakov@bham.ac.uk (M.C.); 2 Jaguar Land Rover Research Department, Coventry CV3 4LF, UK; ttran3@jaguarlandrover.com (T.-Y.T.); nclarke2@jaguarlandrover.com (N.C.)

**Keywords:** radar remote sensing, sonar applications, supervised learning, classification algorithms, artificial neural networks, multilayer perceptron, parameter extraction, sensor fusion

## Abstract

In this paper we shall discuss a novel approach to road surface recognition, based on the analysis of backscattered microwave and ultrasonic signals. The novelty of our method is sonar and polarimetric radar data fusion, extraction of features for separate swathes of illuminated surface (segmentation), and using of multi-stage artificial neural network for surface classification. The developed system consists of 24 GHz radar and 40 kHz ultrasonic sensor. The features are extracted from backscattered signals and then the procedures of principal component analysis and supervised classification are applied to feature data. The special attention is paid to multi-stage artificial neural network which allows an overall increase in classification accuracy. The proposed technique was tested for recognition of a large number of real surfaces in different weather conditions with the average accuracy of correct classification of 95%. The obtained results thereby demonstrate that the use of proposed system architecture and statistical methods allow for reliable discrimination of various road surfaces in real conditions.

## 1. Introduction

Modern vehicles sense their surroundings with such techniques as radar, sonar, LIDAR, GPS, and video cameras [1]. A lot of progress in developing of advanced control systems has been achieved during the last years, so that adaptive cruise control, lane departure warning system, parking assist system, blind spot monitoring, traffic sign recognition, and collision avoidance systems have become standard in modern cars. Such systems provide a more comfortable driving experience by relieving the driver of routine tasks and working in background to keep him safe. There is a trend towards a use of assistance systems also in cheaper vehicles which are sold in higher quantities and require affordable sensors.

However no systems have been developed that allow to remotely recognize the type of road surface and warn the driver or an automated system of the potential hazards associated with the possible loss of control on slippery roads or on off-road surfaces (ice, snow, water, mud, wet grass, etc.) Sub-zero temperatures are extremely dangerous situation for driving, especially in cases when rain or snow are falling on the wet asphalt and solidify forming black ice [2].

The fact that this problem has not been resolved shows its complexity. The use of different types of sensors for surface recognition and detection of low-friction spots has been investigated in many studies and we will provide a review of these papers. Due to the fact that the scattering points of the surface are distributed randomly and measurement results vary within a certain range, this problem can be solved only by statistical methods.

The analysis of the use of LIDAR and optical technologies for road surface recognition can be found in [3]. A serious limitation of optical and partly LIDAR sensors is the loss of performance under adverse weather conditions due to scattering of raindrops, snow, etc. [4]. Optical sensors, moreover, have restricted usability at night and at low sun inclination. Therefore these techniques have limited application to road surface classification.

Information from the short-range sensors mounted on the vehicle can be used to detect the type of road pavement. For example, analysis of the acoustic tire noise profile can provide good results in determining asphalt status [5,6,7]. However such methods do not allow remotely detecting the potentially dangerous surface and therefore taking preventive measures.

Along with other sensor technologies, ultrasonic sensors are widely used in automotive applications; a short review of the papers on surface classification using sonar is presented in [8]. The objectives of the papers [9,10,11,12,13,14,15,16] were to classify surfaces using ultrasonic signals, primarily for use in robotics. In the case of robotic applications the task of classification is simplified comparing with automotive, as robots speed is usually low and the distance is limited to a few meters. This limitation reduces the requirements for the system power and for the resistance to atmospheric phenomena, such as snow, rain, fog, etc.

In [9] time delay spectrometry and neural networks were used to identify surfaces by their frequency response characteristics. The features have been extracted for separate regions of the echo signal. The resultant system achieved almost 100% probability of correct recognition of a set of 12 indoor surfaces with different periodic profiles. In the current research we have improved the segmentation approach proposed in [9] and the developed method will be described in this paper.

A broadband, frequency modulated sonar sensor was effectively used to extract information about the geometry and types of certain surfaces in [10]. In [11,12] a discrimination of different kinds of surfaces has been discussed. The features, extracted from sonar signature, allow distinguishing between five selected indoor and outdoor surfaces with success rate approaching 100%. The application of the Energy-Duration-Range (ENDURA) method for surface classification by its roughness is described in [13,14]. This method was able to identify different surfaces by matching measured echo-energy and echo-duration maps with the templates. Sonar performance in distinguishing between surfaces with random and periodic textures has been studied in [15]. In [16] the neural network has been trained to differentiate between wood, carpet, curtain, ceiling, and water covered surfaces. The results of correct recognition were between 92% and 98%.

The theory of ultrasound propagation in the air and reflection of ultrasonic signals from surfaces with different roughness is well developed and allows simulating the reflected signal [17,18,19].

The use of microwave technologies for surface classification was investigated in [20,21,22,23,24,25,26,27,28,29,30,31,32]. The extensive reviews of the papers on surface classification using radar techniques can be found in [20,21]. Already in the earliest studies the use polarimetric radar has been proposed [22], since the polarization of the reflected signal is dependent on the properties of surfaces [23].

In papers [24,25,26,27] the properties of radar signal reflected from typical road surfaces, including snow and ice covered, have been studied. Special attention was paid to the measurement of backscattering ratios for different polarizations which has shown good results in detecting low-friction areas. The obtained results demonstrate that water, ice, and snow on roads change microwave signals scattering properties, creating the potential for surface recognition.

In [28] was shown that multifrequency radar can be used for snow and ice detection on the road in laboratory conditions. The objective of the papers [29,30] was the detection of areas on asphalt, covered with water, snow, and ice. The use of dual-channel 94 GHz polarimetric radar and Bayesian classifier allowed reliable surface classification in laboratory conditions with the accuracy of above 94%.

Characterization of the scattering behavior of surfaces is a complex task which requires knowledge of the effective dielectric constants related to the road pavement as well as to water, snow, ice or their mixture. In [31] the propagation, reflection and diffraction of 77 GHz radar signal were investigated theoretically and validated by measurements of realistic road surfaces and geometries. Polarimetric backscatter behavior of concrete and asphalt road surfaces was investigated in [32]; good agreement was observed under variety of conditions between the measured and predicted backscatter responses.

In the reviewed papers only few surfaces have been investigated by analysis of specific backscattering properties, so that main attempts have been made to differentiate between dry asphalt and asphalt covered with water, ice or snow. For off-road driving control a wider range of surfaces is challenging and should be investigated, such as gravel, sand, mud, and grass-covered roads. In this paper the extended set of road surface types is under analysis.

In our study, we combine polarimetric radar and sonar techniques for surface classification. The advantage of sonar is its low cost and abundance at a sufficiently high ability to classify surfaces. Its limitations related to the dependence on the environmental conditions (temperature, pressure, and humidity) and the signal absorption by snow, rain, etc. The radar has much greater range and it is resistant to adverse weather conditions. However, polarimetric radar is more complex and expensive than sonar.

In order to achieve reliable classification, the training database should be statistically representative. This requires a large number of measurements for each type of surface at fixed measurement parameters (environmental conditions, incidence angle, signal frequency, transceiver height, etc.), then a reliable surface identification technique can be established based on statistical classification methods.

In this paper, we will discuss a novel approach which addresses the surface classification process. The first step of the developed procedure is the extracting of features from radar and sonar backscattered signals separately for each segment of the illuminated surface. Next, we utilize principal component analysis (PCA) to generate principal components and select the most significant of them. On the last stage the procedure of supervised classification is applied to feature data. We will discuss the classification techniques involved and present the results that have been achieved. The main attention will be paid to artificial neural networks (ANN) that allow an overall increase in correct classification rate. Furthermore, in this paper the factors that influence the accuracy of surface recognition while driving will be analysed. This issue wasn’t highlighted in the reviewed papers.

The remainder of this paper is organized as follows: in Section 2 sonar and radar signal backscattering from surfaces with different roughness is analyzed. In Section 3 the proposed method of surface classification is described. The performance of supervised classification methods is investigated in Section 4 and in Section 5, where the use of multi-stage ANN is discussed. Analysis of the impact of vehicle movement on surface recognition is presented in Section 6 and, finally, the conclusions are formulated in Section 7.

## 2. Signal Backscattering

The theoretical aspects of propagation and reflection of microwaves and ultrasonic signals given in this section are based on known and described in the literature laws. We felt it necessary to bring the basic relations, as it allows us to better understand the design of the system, selection of signal features, choice of surface classification methods, and the operation of the system under different conditions.

### 2.1. Sonar Signal Backscattering

When dealing with the uniform surfaces three parameters defining specific scattering must be considered: the angle of the incidence of ultrasonic wave, the signal bandwidth, and the roughness and the texture of the surface. The surface roughness is defined by the relationship of the surface irregularities and the wavelength of the incident signal. The backscattered energy decays with the grazing angle (distance) more slowly for rough surfaces, than for smooth surfaces [13,14]. Typical sonar signatures are noisy due to interference of reflections across the surface and from air currents [12].

The development of theoretical backscattering model requires knowledge of surface properties, which may vary with different weather conditions, temperature, and atmospheric pressure [17].

**Figure 1:**
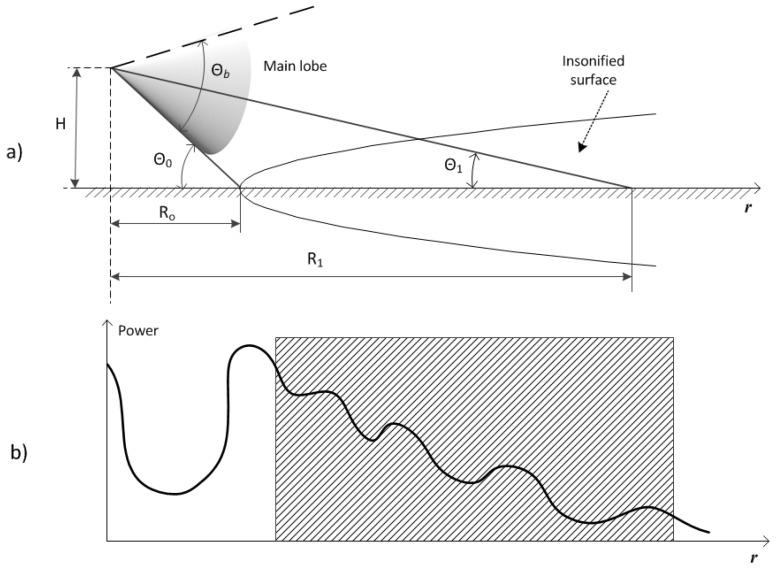
Surface identification in front of a vehicle: (**a**) measurement setup, (**b**) power of backscattered signal; the shaded area represents the range of surface identification.

In Figure 1 the scheme of surface identification in front of the vehicle is shown, where $\theta_{b}$ is the antenna beamwidth, *H* is the antenna height over ground, and *R*_1_ is the distance to the surface at the angle of $\theta_{1}$. The minimum distance to the surface is *R*_0_, it corresponds to the angle of $\theta_{0}$.

For the ultrasonic sensor we can express the amplitude of the received sonar signal in terms of these quantities [10]:

(1) $$A(r)=A_{0}G(r)\;Q(\theta )\;\rho_{b}$$

where $A_{0}$ is a constant, G(r) is the transmission gain function which attenuates with the distance to the surface *r*, Q(θ) is the directivity of the receiver and transmitter pair at grazing angle θ (r), and $\rho_{b}$ is the backscattering coefficient characterizing roughness and texture of the surface.

The expression for the normalized directivity pattern of the plain circular piston transducer can be derived from [18]:

(2) $$Q(\theta )=\left|\frac{2\;J_{1}\left[k_{air}\;a\cdot \sin(\theta -\theta_{1})\right]}{k_{air}\;a\cdot \sin(\theta -\theta_{1})}\right|$$

where $J_{1}$ is the first-order Bessel function and  a is the radius of the transmitting and receiving elements. The wave number $k_{air}$ is the ratio between the angular frequency of ultrasonic waves and the speed of ultrasonic waves in the air $k_{air}={2\pi \;F_{0}/c}_{air}$, where $F_{0}$ is the transmitted signal frequency and $\;c_{air}$ is the speed of ultrasonic wave. The increasing temperature and humidity lead to a considerable echo signal damping [19].

The backscattering coefficient can be derived by considering the physics of propagation of ultrasonic wave reflected from a surface. A surface can be characterized as random Gaussian process and described by three parameters $\left(\rho_{S},\;T_{S},\sigma_{S}\right)$, where the standard deviation of surface heights is $\sigma_{S}$, the reflection coefficient is $\rho_{S}$, and the correlation length is $T_{S}$ [13]. For such a surface, the backscattering coefficient is given as [10]:

(3) $$\langle \left|\rho_{b}\right|\rangle =\sqrt{\frac{\pi }{S}}\;\frac{\eta \;\rho_{S}}{2k_{air}\cos^{3}\theta }e^{-\tan^{2}\theta \;\cdot \eta^{2}/2}$$

where *S* is the area of the reflecting surface, $\eta =T_{S}/\sigma_{S}$ is the roughness parameter, and $\rho_{S}$ is the equivalent reflection coefficient for scattering in the specular direction.

The transmission gain function G(d) in the far field depends on two factors: one is exponential factor, due to the absorption in the propagation medium (air damping), and the other is the inverse second power propagation loss:

(4) $$G(r)=G_{0}\frac{W(r)}{r^{2}}\;e^{-2\alpha \;r}$$

where coefficient $G_{0}$ depends only on the reflector properties and α is the atmospheric ultrasound attenuation coefficient, which can be taken from [19]. Attenuation is proportional to the square of signal frequency and depends on temperature and humidity. For 40 kHz signal α≈1.321 dB/m at air temperature of 20 °C and relative humidity of 60%. In (4) W(r)  accounts for the increase of the insonified area with the distance from the transducer and represents the length of a swathe sector at a distance *r*. It can be calculated for given $\theta_{b},$ $\theta_{1}$, and *H* by solving the equation of intersection of the surface plane with the cone, which constitutes the main lobe.

Analysis of Equations (1)–(4) as well as experimental data allows drawing conclusions about the reflected signal features. These features are related to the signal power and waveform, especially the rate of signal attenuation with the distance and the shape of the signal envelope.

### 2.2. Microwave Signal Backscattering

The overall polarimetric backscatter response of a road surface is composed of volume and surface scattering components [32]. The specification of volume scattering requires knowledge of the effective dielectric constants of the surface and the surface covering substance such as water, ice or snow. The surface scattering plays an important role when the surface roughness parameters are comparable to the radar signal wavelength.

Polarization of reflected electromagnetic wave undergoes changes. Therefore if the surface is illuminated, for example, by a vertically polarized signal, it reflects both vertically and horizontally polarized waves. This depolarization is determined mainly by the dielectric constant of the surface material and the surface roughness. In general, the properties of a surface can be described by scattering matrix of the scattering target [23]:

(5) $$S=\left[\begin{array}{l} S_{vv}\;S_{vh}\\ S_{hv}\;S_{hh} \end{array}\right]$$

In this and in the following expressions the first index refers to the transmitted signal polarization and the second index refers to the received signal polarization. Scattering amplitude *S_ij_* can be expressed as:

(6) $$S_{ij}=\left|S_{ij}\right|\cdot e^{i\varphi_{ij}},\;i,j=v\;or\;h,$$

where |*S_ij_*| is a magnitude and *ϕ_ij_* is a phase angle. Means of backscattered polarized signal magnitudes (|*S_vv_*|, |*S_hh_*|, |*S_vh_*| and |*S_hv_*|) are Rayleigh-distributed [23] and related to the surface geometrical and dielectric properties. For most surfaces the phase angles (*ϕ_vv_*, *ϕ_hh_*, *ϕ_vh_*, and *ϕ_hv_*) and cross polarized phase angles (*ϕ_x1_*= *ϕ_hv_ − ϕ_vv_* and *ϕ_x2_*= *ϕ_vh_ − ϕ_vv_*) are uniformly distributed over [*−π, π*] and contain no information about surface parameters. The co-polarized phase angle (*ϕ_c_*= *ϕ_hh_ − ϕ_vv_*) is target-dependent and depends on the signal parameters, such as grazing angle and wavelength, and on the surface parameters, such as roughness and dielectric constant. In addition, microwave radiation is very sensitive to the presence of water in the medium through which it passes.

The number of influential physical parameters on radar responses of surfaces such as surface dielectric constant, roughness, wetness, density, surface cover, etc. is rather large, which makes the development of theoretical backscattering model rather difficult. It requires a different approach to the analysis of various surfaces [32]. Therefore concerning the implementation of surface recognition system the most practical approach is the statistical classification method.

## 3. Method of Surface Classification

### 3.1. Surface Classification Procedure

The goal of our study is to trace the difference in relative parameters of backscattered signal from various types of surfaces under other equal conditions (frequency, polarization, grazing angle). The procedure of surface classification involves a three-stage process: segmentation, feature extraction and classification.

In most studies discussed in Section 1 (Introduction), the features were extracted from the reflected signal related to the entire illuminated footprint. However, as can be seen from the analysis of the Equation (1), the attenuation of the ultrasonic echo signal with the distance depends on the surface properties. In our experiments the similar dependence was also observed for microwave signals. Therefore it is desirable to obtain not only the average values of the features, but also their variation with increasing distance, or what is the same, with decreasing grazing angle. The angular dependency of backscattered signal can be considered as the characteristics of surface roughness.

During segmentation the illuminated footprint is divided into individual strips of a certain width (swathes), each is located equidistantly from the antenna. The width of each swathe and consequently their number depends on the range resolution of radar or sonar. The features are extracted for each swathe (or for their combination). The number of extracted features can be easily increased with increasing number of processed individual swathes. Let the number of swathes is *N_S_*, the number of independent backscattered signals is *N_B_* and the number of features for *i*-th signal and *j*-th swathe is *F_ij_*. In this case the maximum number of features equals:

(7) $$M_{F}=\sum_{j=1}^{N_{S}}\sum_{i=1}^{N_{B}}F_{ij}$$

The increase in the number of features leads to higher classification accuracy but also to an increase in the computational complexity of the algorithm.

Classification involves identifying the surfaces in question. In this paper only supervised classification algorithms (classifiers) will be discussed, where a set of features and, therefore, cluster domains are initially to be defined [33]. Under the cluster domain we understand a set of obtained signal samples belonging to the same surface. During the training stage the database of signal features is accumulated and the sub-set of features is selected by the criterion of the best classification algorithm performance. During the classification stage the decision on belonging of each measurement to a particular cluster is made based on the extracted signal features.

The feature data, used for training, may consist of correlated information. Correlated data lead to the classification algorithms with low generalization capability [34]. Principal Component Analysis (PCA) technique [35] allows for eliminating correlation at the sample data before they are being presented to classification algorithm. The PCA method allows reducing the dimensionality of input space, improving the classifier’s performance and reducing the training time [36]. In order to eliminate the possible correlation of the training data, MATLAB Neural Network Toolbox PCA pre-processor [37] has been used.

### 3.2. Experiment Setup

**Figure 2:**
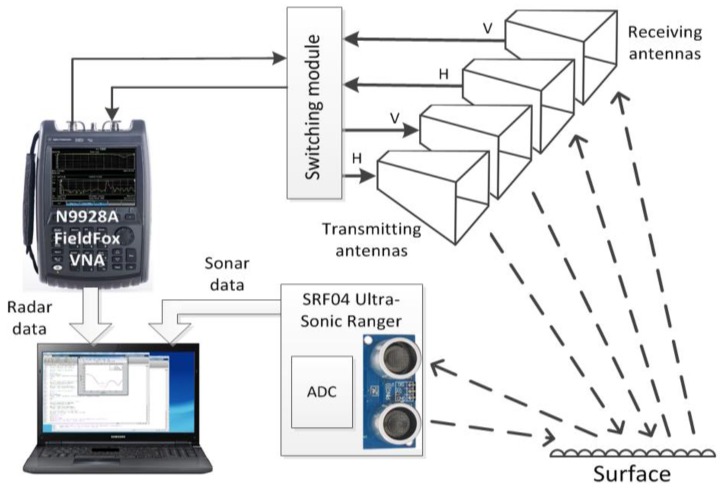
Block diagram of the measuring system.

In this paper we will consider surface classification based on the system, consisted of polarimetric radar and sonar [21], which is shown in Figure 2. The sonar beam pattern is conical with the width of the beam of 55°. Antenna beamwidth was chosen to insonify the entire width of the lane, while avoiding reflections from roadside objects. Sonar frequency was within the automotive ultrasonic sensors frequency range: the echo was measured at 40 kHz (wavelength 8.6 mm), signal range resolution is 35 mm. The installation height of the transceiver was 0.65 m and the grazing angle was 10°. At smaller angles the reflected signal becomes too weak for reliable classification of surfaces; higher angles reduce the effective range of the system.

Forward-looking monostatic radar has been developed for the experiment; it was based on FieldFox N9918A network analyzer (Keysight Technologies, Inc., Santa Rosa, CA, USA). Two coherently interconnected transmit and two receive antennas with orthogonal polarization allowed to realize a coherent polarimeter. Four horn antennas with a beamwidth of 60° have been manufactured to operate at 24 GHz. The polarization of the receiving and transmitting antennas can be controlled by the switching module.

**Figure 3:**
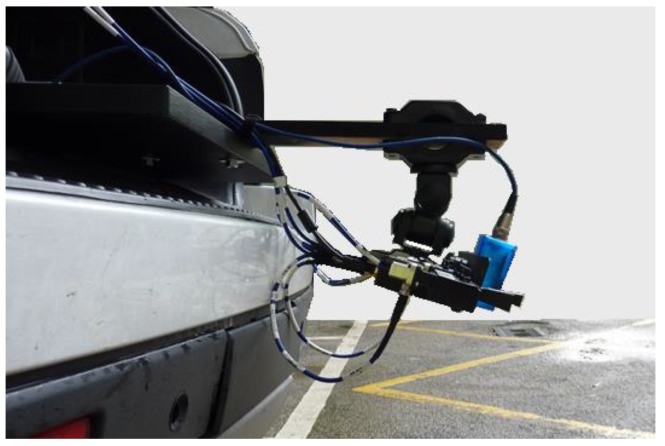
Sonar and radar mounted on a vehicle.

The microwave frequency range has been established in accordance with the general requirements for the bandwidth and frequency of automotive radars [38,39]. The signal bandwidth was 200 MHz at 24 GHz central frequency that corresponds to range resolution of 0.75 m. Photo of sonar and radar mounted on a vehicle is shown in Figure 3.

The backscattered sonar and radar signals were measured after path loss compensation. We have considered only propagation loss, which is proportional to the square of the distance between the transmitter and the receiver. In the developed monostatic system path loss in decibels can be calculated using the formula *L =* 20log_10_*(d)*, where *d* is two-way distance between the transceiver and the surface. If we take a distance of 1.5 m for the initial count, then to compensate for the losses we must add, for example, approximately 8.5 dB to the signal reflected from the surface at a distance of 4 m. In order to get rid of unwanted reflections (sidelobes, air fluctuations and other obstacles, range gating was used. Two swathes were allocated of the full range from 1.5 m to 4.0 m: the first at the distance from 1.5 m to 2.5 m and the second at the distance from 3.0 m to 4.0 m. The selection of these swathes was determined by the characteristics of the reflected signals. Different surfaces have different rates of attenuation of the reflected signal, so it is necessary to analyze the magnitude of the reflected signal at the beginning and the end of range. Moreover, the length of the swath should exceed the radar range resolution, i.e., 0.75 m.

The full list of obtained features consists of 34 different values [21]. Since the features have different influence on the performance of the algorithms, it is important to determine the most influential ones defining performance of classification algorithm by its accuracy. Our approach to the optimal choice of features was based on sequential forward selection method: we added features one by one, at each step adding the one that decreases the error the most, until any further addition did not decrease the error. The list of thirteen features, which provide the best surface identification, is presented in Table 1.

On the next stage we applied PCA algorithm to the remaining features and generated a new table with the same number of principal components as the number of features. As a result of PCA data transformation, first principal components have the largest variance. In case of ANN, six most significant components provide classification accuracy of about 87%; further including of seven next components leads to increase in accuracy of 5%. In the considered system all 13 components were used for classification.

The mean power and the standard deviation of the backscattered radar signal were measured at four different combinations of transmitted and received antennas polarizations: vertical-vertical (VV), vertical-horizontal (VH), horizontal-horizontal (HH), and horizontal-vertical (HV).

In addition to the mean power and the standard deviation of the echo signal envelope, ultrasonic signal features include the signal power and duration above the threshold. The threshold value was based on the mean amplitude of the ultrasonic signal. In the case of radar the threshold was not used, since microwave signal range resolution is low in comparison with the length of the range gate. The mean power of the reflected signal is defined by the surface reflection coefficient and its dependence on distance and, in the case of the radar, on signal polarization. The standard deviation of the signal envelope is determined by the roughness and texture, which is also one of the characteristics of the surface. During the development of the system we have seen that in many cases the analysis of the signal power and duration above the threshold can significantly improve the system performance.

In this study, we did not aim to analyze a large number of statistical classification algorithms. We were guided by a practical approach, namely, how to use simple methods to ensure a reliable surface identification. Therefore, some of the known methods, such as support vector machines, remained outside of this work.

In the paper four common classification tools will be considered for surface identification. Two methods belong to the category of parametric methods: maximum likelihood estimator (MLE) and minimum distance classifier (MDC). In parametric methods we assume that the distribution of samples in cluster is known; it is normal in the case of MLE and spherical in the case of MDC with Euclidean (MDC-E) or the Mahalanobis (MDC-M) distances. The test point is classified as belonging to a class for which the distance to the center of the cluster is minimal.

In nonparametric KNN method each observation belongs to a cluster with the nearest Euclidean distance to its *k_n_* neighbors. The shortcoming of KNN method is that all the training data samples must be retained. This might lead to problems of computer storage and can require large amounts of processing to evaluate the surface for new input values. The value of *k_n_*, which is usually much smaller than the number of test samples, defines the performance of the method. When *k_n_* is too small, single instances have a large effect resulting in decreased accuracy of the method. When *k_n_* is too big, the computational complexity and the bias increase [33].

The second considered nonparametric method was a feed forward ANN based on MLP, composed of several layers of nodes with unidirectional structure.

The collected database consists of more than 2800 feature vectors; the length of each vector is 13, which corresponds to the number of features. The database for each type of surface is composed of approximately 200 measurements which were collected at three or four different locations. Fourteen various types of surfaces have been included into the database. Some of them represent surfaces, typical for driving in summer conditions: dry asphalt (AD), dry bitumen (BD), dry gravel (VD), dry grass (GD), wet grass (GW), dry ground (DD), wet ground (DW), and dry sand (ND). Wet grass is an important type of surface, because it is very slippery. We have examined different types of winter road surfaces, including dry snow (SD), wet snow (SW), snow covered with crust (SI), clear ice (ID), snow on ice (IS), and asphalt, covered with compacted snow (AS).

In connection with the objectives of this study, we focused on a variety of surfaces, typical for off-road driving. Distinctions between representative surfaces are defined by different electro physical parameters (i.e., dielectric permittivity, thermal conductivity, etc.) and shape factors (roughness, structure, etc.). However only combined contribution of all these factors could be used to get reliable classification in a statistical manner.

**Figure 4:**
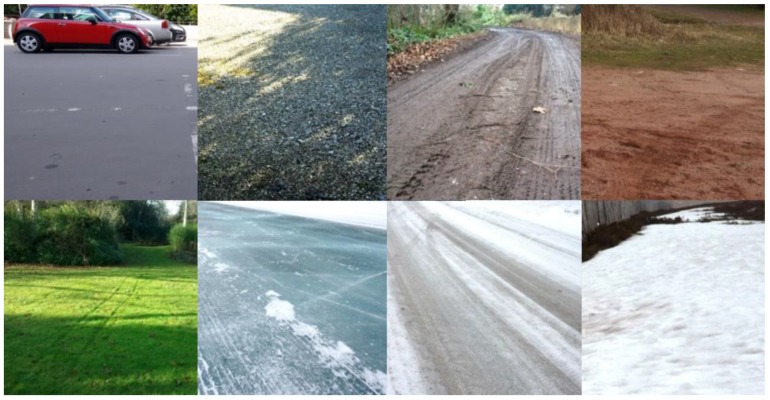
Examples of investigated surfaces: top row (left to right): bitumen, gravel, ground, sand; bottom row (left to right): grass, ice, asphalt covered with compacted snow, wet snow.

Most types of surfaces were investigated in spring, summer and autumn seasons near Birmingham and Coventry, UK. The investigated asphalt had low wear and average roughness; bitumen was very smooth; the average size of gravel pebbles was from 1 to 2 cm; grass has been cut. Winter tests were carried out in the north of Scotland and in Lapland (Sweden). Smooth thick ice was investigated on a frozen lake; the thickness of deep dry snow exceeded 5 cm. Photos of some investigated surfaces are shown in Figure 4.

## 4. Experimental Results

The object of classification is to design a rule that assigns objects (experimental results), to one of the classes (surfaces), on the basis of feature vectors of those objects. The simplest performance metrics can rely on computing the classifier’s predicted classes ${\hat{y}}_{1},\;{\hat{y}}_{2},\;\ldots {\hat{y}}_{n}$ for the *p* test patterns with their true labels $y_{1},\;y_{2},\;\ldots y_{n}$, where *n* is the number of classes. The primarily statistics of interest are misclassification counts. True Positives (*TP*) Together with False Positives (*FP*) form confusion matrix (Table 2).

In machine learning and statistics, the most widely used summary of the above matrix is the error rate (*Er*), which is simply the total misclassification count divided by the number of examples [33]. In this paper we will also use true positive rate (or accuracy), which is the percent of correct classifications Tr=1−Er and can be derived from the confusion matrix:

(8) $$Tr=\frac{1}{p}\sum_{i=1}^{n}TP_{i}$$

In the problem considered here classifiers are learned (trained) and tested on a finite training and testing data sets. More training data give better generalization (ability to classify unseen data) but more test data gives better estimate for the classification error probability. All results reported in this paper were obtained by analysis of data randomly partitioned into two independent sets of the same size: the training and the test sets.

**Figure 5:**
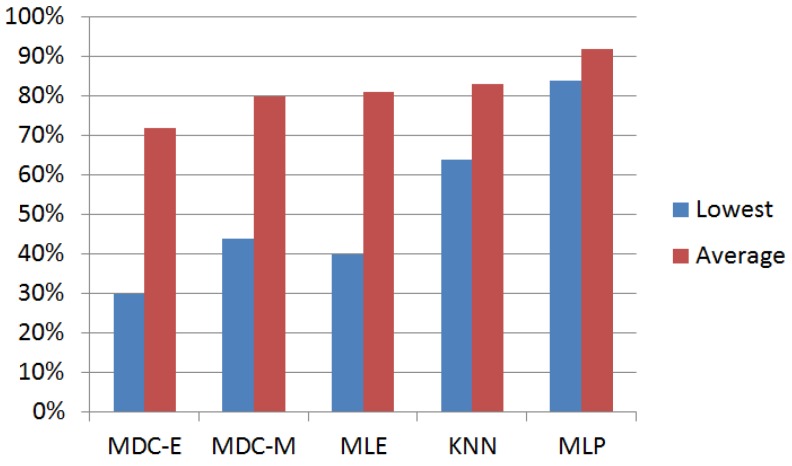
Accuracy of classifiers.

Accuracy (*Tr*), achieved with the use of different classification methods, is presented in Figure 5. As can be seen from the figure, the accuracy of non-parametric methods (KNN and MLP) is better than parametric methods (MDC and MLE). This is due to the fact that assumption of normal feature distribution is incorrect for some types of road surfaces. The optimal value of *k_n_* in our experiments was three. A MLP is a kind of feed-forward ANN model, consisting of three adjacent levels, called the input layer, the hidden layer and the output layer [40].

Two methods stand out from the total number of the methods considered. The most inaccurate is the simplest MDC-E method. MLP, in contrast, shows much better accuracy than other classifiers. Another MLP advantage is a minimal variation of classification accuracy of different surfaces. MLP demonstrated the lowest classification accuracy of 84% in the case of asphalt, covered with snow and ice (AS), while the accuracy of other methods in some cases dropped to 30%–40% (see Figure 5).

In the case of automotive applications, we can be restricted by the speed of a vehicular computer. According to the criterion of computer performance, parametric methods are preferable since they are computationally simpler. Complexity of nonparametric KNN method is proportional to the size of the training set. MLP method requires computationally intensive training and optimization phase. However, the classification process is usually fast and can be implemented on the basis of specialized microcontrollers, such as Arduino boards. Taking into account all advantages of MLP, in the current research we have focused on the use of this method for surface classification.

Results of classification using MLP method are presented in Table 3 in terms of a confusion matrix. Each column of the table represents the recognized surface type, while each row represents the actual surface type. As can be seen from the table, this method provides confident surface identification with the average accuracy exceeding 92%. MLP was trained using Bayesian regularization, the number of nodes in the hidden layer was 13. It should be noted that the network structure was optimized for the case under consideration, when different sets of data were used for training and testing, but collected in static in the same places. In the case of the classification of novel unseen data, the smaller architecture usually allows for better generalization properties. Therefore, in practical implementation of the system, it may be necessary to reduce the number of nodes in the hidden layer. According to our results, at five nodes the average classification accuracy was 88%, which is only 4% less than at 13 nodes. However, with a reduction in the number of nodes to four, the accuracy dropped to 83%.

We can see from the table that the accuracy of differentiation between certain surfaces is significantly lower than the average accuracy. Thus, dry ground in 5% of cases was recognized as wet ground, and in 3% of cases as sand. In general, these three surfaces (outlined by a frame in the Table 3) show increased levels of mutual false positives. This can be explained on the basis of their similar physical properties.

These considerations can be applied to ice, snow on ice, and snow and compacted snow on asphalt (ID, IS, AS), which are outlined by another frame in the Table 3.

As the number of considered surfaces increases, the task of confident classification of similar surfaces becomes more difficult. Therefore in the next section we will consider ANN optimization and, in particular, will examine the efficiency of multi-stage ANN in surface classification.

## 5. ANN Structure Optimization

In this paper we will consider multi-stage ANN for surface classification. In the first stage the ANN is trained to recognize aggregated classes of surfaces. The responses are then presented to another network where the final decision is made. The proposed original classification scheme is based on the general approach described in the literature [41,42,43]. It was tested and its performance is compared with that of a conventional ANN.

**Figure 6:**
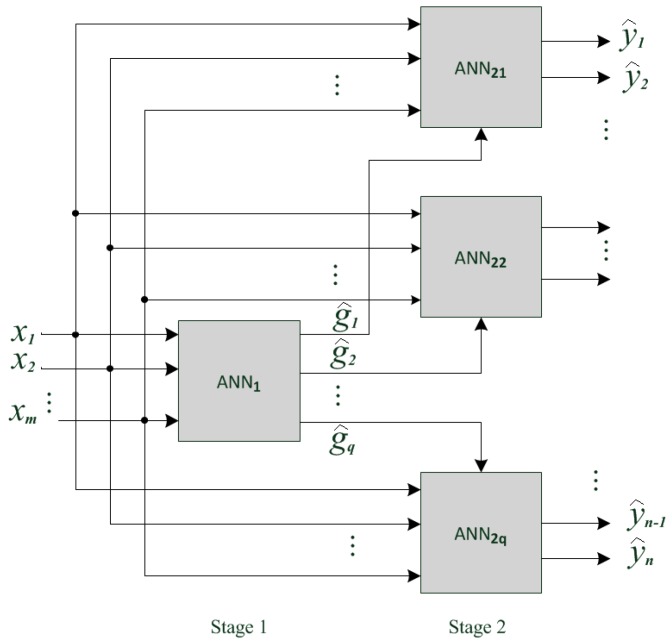
Two-stage ANN structure.

In a conventional ANN the emphasis is put on those training data where performance is poor [42]. The optimized conventional network structure makes it possible to distinguish these difficult cases, but by reducing the overall accuracy. The advantage of the proposed multi-stage ANN is improved performance especially in cases of surfaces with similar features. The multi-stage gives the scalability to the system, because when we add or change one class, we have to train only the affected ANN and it is not necessary to re-train the entire system. The two-stage ANN block-diagram is shown in Figure 6.

In the first phase of multi-stage network development, all surfaces are divided into *q* classes with similar features of backscattered sonar and radar signals. The first stage ANN_1_ should be trained to made classification between classes. Its outputs (${\hat{g}}_{1},\;{\hat{g}}_{2},\;\ldots {\hat{g}}_{q}$) trigger the appropriate second-stage ANN, which should classify the surface within the corresponding class. The number of ANN stages determined by the number of surfaces, its classes and subclasses. In this paper, we will analyze the performance of a two-stage network that is sufficient for a number of investigated surfaces (*n* = 14).

In our research, all ANNs were trained and tested with the same sets of training and test patterns. To obtain the optimum ANN structure, Bayesian regularization training algorithm was used. After the ANNs had been trained, their performances were evaluated based on the test data. In doing so, the test data were first pre-processed using PCA algorithm.

The classifier must be able to classify new unseen data (generalize). Increasing the number of hidden nodes increases the risk of “overfitting,” when statistical model describes a specific example, and not the general law. Overfitting of the training data leads to deterioration of generalization properties of the model and results in its untrustworthy performance when applied to novel measurements. The optimal number of hidden nodes is typically between the number of nodes used for the input and the output layers [44].

The true positive rate of two-stage ANN can be calculated as the ratio of correctly classified surfaces to the total number of test patterns:

(9) $$Tr^{\left(1+2\right)}=\frac{1}{p}\sum_{i=1}^{q}\sum_{j=1}^{n_{i}}TP_{j}{}^{\left(2i\right)}$$

In (9) and the following equations the upper index is related to the corresponding ANN, so is the True Positives on the *j*-th output of the second stage ANN_2i_ (see Figure 6), *n_i_* is the number of ANN2i outputs (number of surfaces in *i*-th class). Similar to (8), the true positive rate of the first ANN stage can be written as:

(10) $$Tr^{\left(1\right)}=\frac{1}{p}\sum_{i=1}^{q}TP_{i}{}^{\left(1\right)}$$

Taking into consideration that True Positives on *i*-th output of the first stage ANN_1_ equals to the number of patterns of the second stage ANN_2i_ (indeed, the signal is supplied to ANN_2i_ only if ANN_1_ decides that the surface belongs to *i*-th class):

(11) $$TP_{i}{}^{\left(1\right)}=p^{\left(2i\right)}=\sum_{j=1}^{n_{i}}TP_{j}^{\left(2i\right)}+\sum_{j=1}^{n_{i}}\sum_{\begin{array}{l} \;k=1,\\ \;k\neq j \end{array}}^{n_{i}}FP_{jk}^{\left(2i\right)}$$

Equation (9) can be rewritten as:

(12) $$Tr^{\left(1+2\right)}=Tr^{\left(1\right)}-\frac{1}{p}\sum_{i=1}^{q}\sum_{j=1}^{n_{i}}\sum_{\begin{array}{l} \;k=1,\\ \;k\neq j \end{array}}^{n_{i}}FP_{jk}{}^{\left(2i\right)}=Tr^{\left(1\right)}-Er^{\left(2\right)}$$

Thus, the true positive rate of considered two-stage ANN equals to the difference between the true positive rate of the first stage and the summary error rate of the second stage. Therefore, in order that such a system will be more effective than a conventional scheme, the accuracy of the first stage should increase substantially comparing with the *Tr* of a single network.

The above considerations have been tested on the basis of the collected data. In order to define classes of surfaces (e.g., find surfaces with similar features) we conducted a stepwise classification, at every step removing from the training database the surface, which was closest to the surface of interest to us. The feature database of the surface of interest was used as a test database. As a result all surfaces were grouped into the following three classes:

Class 1 (C1): ice, snow on ice and compacted snow on asphalt (ID, IS, and AS);
Class 2 (C2): dry ground, wet ground, and sand (DD, DW, and ND);
Class 3 (C3): all other surfaces (AD, BD, VD, GD, GW, SD, SW, and SI).

As can be seen from the Table 3, surfaces constituting Class 1 and Class 2 have increased levels of mutual false positives, from which we can conclude that they have similar features.

In the Table 4 the results of classification using two stages ANN are presented. As can be seen from the table ANN_1_, in the first stage the method provides good separation of classes with $Tr^{\left(1\right)}=98.5\%$. It should be taken into account that classes of surfaces differ in size and therefore have different weights when calculating this result; class C3 makes the greatest contribution. Tables ANN_21_, ANN_22_, and ANN_23_ show the classification results in the second stage. The method provides a reliable identification of surfaces within each class with $Tr^{\left(21\right)}=91.4\%,$ $Tr^{\left(22\right)}=97.9\%\;,$ and $Tr^{\left(23\right)}=97.0\%$.

All ANNs include an input layer with 13 input nodes, a hidden layer, and an output layer with a number of output nodes corresponding to a number of surfaces within this ANN. The number of hidden nodes was 12 in ANN_1_, 6 in ANN_21_ and ANN_22_, and 10 in ANN_23_. The networks have been trained and tested with the same sets of training and test patterns, respectively. As in the case of a conventional ANN structure discussed in Section 4, complexity of individual networks can be reduced without significant loss of accuracy. Thus, reducing the number of nodes in the hidden layers by half, leads to a decrease in the total average accuracy of two-stage ANN by about 3%.

The accuracy $Tr^{\left(1+2\right)}$, achieved with the use of multi-stage ANN, is presented in the Table 5. Comparing these results with the corresponding results from the Table 3, we can see that the average accuracy increased by about 3%. At the same time the classification accuracy of the most difficult to differentiate surfaces in Classes 1 and 2 has increased considerably. For example, snow and ice on asphalt (AS) was correctly identified in 92% cases (increase in 9%); dry ground (DD) was correctly identified in 97% cases (increase in 12%). Moreover, we have achieved a reduction in the spread of classification accuracy of various surfaces. Indeed, in Table 3 the classification accuracy of the various surfaces was between 84% and 100% (mean 92%), while in a multi-stage ANN (Table 5) it varies from 91% to 100% (mean 95%).

Thus, the experimental results confirm the effectiveness of a multi-stage MLP in case of classifying surfaces with similar features.

All results presented in this paper were obtained in the static mode. The measuring system was stationary at the time of each measurement, and moved to a new position upon its completion. For practical realization of surface recognition system it is necessary to analyze the influence of vehicle movement on backscattered radar and sonar signals. This problem is considered in the next section.

## 6. Influence of Vehicle Movement on Surface Recognition

This section provides only a preliminary review of the problems encountered in surface identification from a moving vehicle. It should be noted that the developed system is designed primarily for off-road driving when the speed is usually limited to 20–30 km/h.

Car vibrations, pitch and roll variations, changes in the height of antennas result in increased noise and instrumental errors and therefore in reduction of classification accuracy. In addition, reflections from objects on the roadside, from other cars, and from air fluctuations should be taken into account. In this section we will assess the impact of these factors on the accuracy of surface recognition.

We first consider the effect of vehicle movement that affects the orientation and the vibrations of antennas. Vehicle pitch and roll result in a change of the illuminated surface area. The analysis of the Equation (1) shows that the change in the grazing angle, caused by vehicle pitch $\theta_{P}=\pm \;2.5{}^{\circ}$, leads to a change in backscattered signal power for less than half a decibel. In most of practical cases this influence can be neglected. The robustness of developed surface classification method is due to use of time gating. Irrespective of variations in antennas installation, we analyze the signal reflected by the same surface area.

The roll factor will not have a noticeable effect on the measurement accuracy in the case of axial symmetry of the beam. However, if we want to reduce errors due to pitch and roll, we must consider the known data about the vehicle's position, received from other internal sensors.

Antennas mounted on vibrating vehicle can receive mechanically coupled interface. These vibrations will make additional random error (noise) in the measured data. If the system is a subject to vibration, a special damping fixation should be considered to minimize the transmission of vibration to antennas. Our experiments show that when the sensors are properly mounted, this noise is low in comparison with the signal and can be neglected.

The second problem is associated with reflections from roadside objects (buildings, fences, bushes, etc.). These reflections can be eliminated by time gating [20] and velocity gating. The velocity of the car in relation to the road is known, which suggests using the Doppler effect to separate the backscattering of the road surface from the moving objects [25].

On the assumption that air is stationary relative to the ground, the sonar Doppler shift $F_{D}$ is approximately proportional to the vehicle velocity:

(13) $$F_{D}\approx F_{0}\frac{V}{c_{air}}\cos\;\theta_{1}$$

where *V* is vehicle velocity relative to the ground and $F_{0}$ is the transmitted signal frequency. Velocity gating can be implemented as a band pass filter with a central frequency of $F_{D}+F_{0}$ [25]. The bandwidth should be chosen with the condition to pass only reflections of the surface. A similar expression can be given for the radar, where $c_{air}$ to be replaced with a speed of electromagnetic waves.

The third problem relates only to sonar and associated with the peculiarities of ultrasound propagation in the air. Increasing the temperature by 5° in most cases reduces the power of sonar echo signal by an average of one decibel. Increasing humidity by 20% leads to signal attenuation of 1 decibel at a temperature of 15 °C [19]. Temperature and humidity sensors, which are equipped with modern cars, can be used as environmental data sources and the appropriate correction can be made in the classification algorithm.

**Figure 7:**
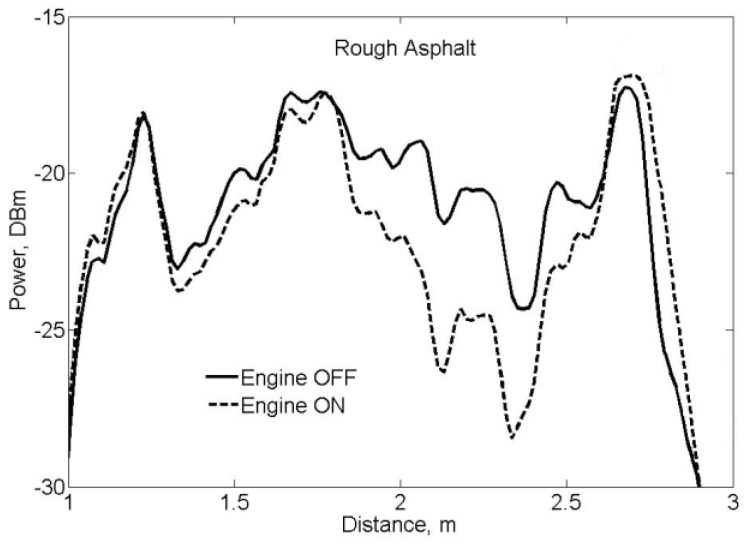
Ultrasonic backscattered signal after path loss compensation.

The influence of absorption and reflection of ultrasonic waves caused by air turbulences is much more difficult to compensate. For example, the vehicle exhaust gases significantly affect the accuracy of sonar measurements. Our results show that if exhaust gases are directed to the asphalt area, which is the object of measurement, the power of the backscattered ultrasonic signal at a distance of two meters decreases by 4 dB (Figure 7).

Radar does not have these drawbacks inherent in sonar. Air temperature, humidity and air fluctuations do not have any noticeable effect on the backscattered microwave signal. Therefore the use of combined ultrasonic and microwave system can considerably improve the accuracy of surface recognition in dynamics.

## 7. Conclusions

In the present study we have investigated a possibility of road surface classification by analyzing the backscattered microwave and ultrasonic signals. The developed system consisted of polarimetric 24 GHz radar and 40 kHz sonar. The recorded signals were processed using statistical classification methods. The analysis of the performance of classifiers was based on the database, which consisted of more than 2800 recorded radar and sonar signals at about 40 outdoor locations.

A set of features for separate swathes of illuminated surface has been defined to be used in classification. These features include values characterizing the power and the waveform of the backscattered signal: mean power, power above the threshold, duration above the threshold, and standard deviation of the signal envelope.

Four common methods of supervised classification have been applied for distinguishing between the fourteen types of surfaces of interest: asphalt, grass, gravel, sand and bitumen, dry, wet, and covered with snow and ice. These methods include minimum distance classifier, maximum likelihood estimator, nearest neighbor method, and artificial neural network based on multilayer perceptron.

Our results show that the analysis of the characteristics of reflected ultrasonic and microwave signals allows distinguishing between different road surfaces under stationary conditions. The use of multi-stage ANNs can be especially helpful in case of classifying surfaces with similar features. The proposed technique was tested for recognition of a large number of real surfaces under different weather conditions with an average accuracy of correct classification of 95%.

The measurement error caused by air fluctuations may considerably deteriorate the performance of the sonar, however, the system consisting of radar and sonar has increased resistance to adverse weather conditions.

## Figures and Tables

**Table 1:** The List of Signal Features.

Signal Features	Swathe 1 (1.5 m–2.5 m)	Full Range (1.5 m–4.0 m)	Swathe 2 (3.0 m–4.0 m)
Mean power	Sonar, Radar VV	Sonar, Radar VV, VH, HV, HH	Sonar, Radar VV
Standard deviaton	-	Sonar, Radar VV	-
Power above the threshold	-	Sonar	-
Duration above the threshold	-	Sonar	-

**Table 2:** Confusion Matrix.

Actual	Predicted Class			
Class	${\hat{y}}_{1}$	${\hat{y}}_{2}$	…	${\hat{y}}_{n}$
$y_{1}$	$TP_{1}$	$FP_{12}$	…	$FP_{1n}$
$y_{2}$	$FP_{21}$	$TP_{2}$	…	$FP_{2n}$
…	…	…	…	…
$y_{n}$	$FP_{n1}$	$FP_{n2}$	…	$TP_{n}$

**Table 3:** Accuracy of MLP Method (in percent).

Surface	AD	BD	VD	GD	GW	DD	DW	ND	SD	SW	SI	ID	IS	AS
AD	**96**	0	2	2	0	0	0	0	0	0	0	0	0	0
BD	0	**100**	0	0	0	0	0	0	0	0	0	0	0	0
VD	1	0	**95**	4	0	0	0	0	0	0	0	0	0	0
GD	4	0	2	**94**	0	0	0	0	0	0	0	0	0	0
GW	0	0	3	0	**95**	0	0	0	0	2	0	0	0	0
DD	1	0	0	0	0	**85**	3	5	0	0	0	0	3	3
DW	0	0	0	0	0	2	**90**	4	0	0	2	0	1	1
ND	0	0	0	0	0	0	5	**94**	0	0	0	0	0	1
SD	0	2	0	0	0	0	0	0	**98**	0	0	0	0	0
SW	1	0	3	7	3	0	0	0	0	**86**	0	0	0	0
SI	2	0	0	0	0	0	0	0	0	0	**97**	0	0	1
ID	0	0	0	0	0	0	0	1	0	0	0	**90**	3	6
IS	1	0	0	0	0	0	0	2	0	0	0	2	**90**	5
AS	0	0	0	0	0	2	0	0	0	0	2	6	6	**84**

**Table 4:** First and Second Stage Accuracy (in percent).

	**ANN_1_**				**ANN_21_**					**ANN_22_**			
Class	C1	C2	C3		Surface	ID	IS	AS		Surface	DD	DW	ND
C1	**97.4**	2.3	0.3		ID	**91**	6	3		DD	**97**	3	0
C2	0.0	**93.0**	7.0		IS	0	**93**	7		DW	2	**97**	1
C3	0.2	0.3	**99.5**		AS	3	6	**91**		ND	0	0	**100**
					**ANN_23_**								
		Surface	AD	BD	VD	GD	GW	SD	SW	SI			
		AD	**99**	0	0	1	0	0	0	0			
		BD	0	**98**	0	0	0	2	0	0			
		VD	0	0	**96**	2	0	0	2	0			
		GD	4	0	2	**94**	0	0	0	0			
		GW	0	0	2	0	**98**	0	0	0			
		SD	0	1	0	0	0	**99**	0	0			
		SW	1	0	1	1	4	0	**93**	0			
		SI	0	0	1	0	0	0	0	**99**			

**Table 5:** Multi-Stage ANN Accuracy of Classification (in percent).

Surface		Surface		Surface	
AD	99	DD	97	SI	99
BD	98	DW	97	ID	91
VD	96	ND	100	IS	91
GD	94	SD	99	AS	93
GW	98	SW	93	Average	95
